# The Biological Axis of Protein Arginine Methylation and Asymmetric Dimethylarginine

**DOI:** 10.3390/ijms20133322

**Published:** 2019-07-06

**Authors:** Melody D. Fulton, Tyler Brown, Y. George Zheng

**Affiliations:** Department of Pharmaceutical and Biomedical Sciences, College of Pharmacy, University of Georgia, Athens, GA 30602, USA

**Keywords:** ADMA, PRMT, arginine methylation, nitric oxide, metabolism

## Abstract

Protein post-translational modifications (PTMs) in eukaryotic cells play important roles in the regulation of functionalities of the proteome and in the tempo-spatial control of cellular processes. Most PTMs enact their regulatory functions by affecting the biochemical properties of substrate proteins such as altering structural conformation, protein–protein interaction, and protein–nucleic acid interaction. Amid various PTMs, arginine methylation is widespread in all eukaryotic organisms, from yeasts to humans. Arginine methylation in many situations can drastically or subtly affect the interactions of substrate proteins with their partnering proteins or nucleic acids, thus impacting major cellular programs. Recently, arginine methylation has become an important regulator of the formation of membrane-less organelles inside cells, a phenomenon of liquid–liquid phase separation (LLPS), through altering π-cation interactions. Another unique feature of arginine methylation lies in its impact on cellular physiology through its downstream amino acid product, asymmetric dimethylarginine (ADMA). Accumulation of ADMA in cells and in the circulating bloodstream is connected with endothelial dysfunction and a variety of syndromes of cardiovascular diseases. Herein, we review the current knowledge and understanding of protein arginine methylation in regards to its canonical function in direct protein regulation, as well as the biological axis of protein arginine methylation and ADMA biology.

## 1. Introduction

Higher-order organisms (e.g., mammals) generally share conserved or similar chemical mechanisms of bimolecular synthesis and metabolism in comparison to lower-order organisms such as prokaryotes. However, a remarkable distinction in the cell biology of higher-order organisms is the complexity of regulation of diverse biological processes such as gene expression, cell cycle, central metabolism, and signal transduction. Sophisticated regulatory mechanisms and networks are present in eukaryotic cells to control or modulate cellular programs for development, differentiation, survival, and adaptation. Such complex mechanisms of regulation include gene fusion/translocation, epigenetics, RNA splicing, protein-protein interaction, protein-nucleic acid association, and post-translational modifications (PTMs) of proteins. Especially, a myriad of post-translational modifications (PTMs) (e.g., phosphorylation, glycosylation, acetylation, methylation) allow for ON/OFF-switching or fine tuning of protein-controlled cellular processes in response to intracellular and extracellular signals. Among the various PTMs, arginine methylation is a widespread PTM that is conserved in all eukaryotic organisms, from yeasts to humans [1,2,3]. The frequency of the arginine methylome is parallel to the corresponding phosphoproteome and ubiquitylome cells [2]. About 0.5–1% of the total arginine residues in the cellular proteome are methylated and have a slow turnover, likely to confer lasting functional properties to proteins [4,5].

**Figure 1:**
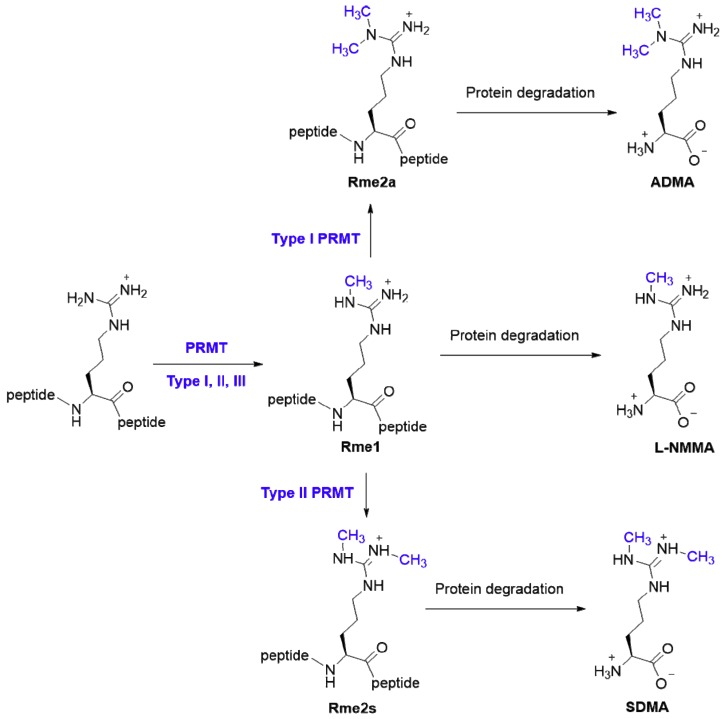
The biological axis of protein arginine methylation and formation of methylarginine small molecule metabolites. Type I, II, and III PRMTs catalyze the initial monomethylation of an arginine residue at the terminal guanidinium nitrogen. Subsequently, type I and II can catalyze a second arginine methylation reaction to form the asymmetric and symmetric dimethylated arginine residues, respectively. Protein degradation of arginine methylated proteins produces the ADMA, l-NMMA, and SDMA metabolites.

Arginine residues can be methylated on the guanidinium nitrogen atoms in three different states—*N^G^*-monomethylarginine (Rme1), asymmetric *N^G^*,*N^G^*-dimethylarginine (Rme2a), and symmetric *N^G^*, *N’^G^*-dimethylarginine (Rme2s) (Figure 1) [3,6,7,8]. Reported results on the stoichiometric abundance of different methylation states, although varying at different degrees, generally support that monomethylated arginine is more abundant than dimethylated arginine sites in proteins. Dilworth and Barsyte-Lovejoy analyzed the number of arginine methylation sites in the Phosphosite database by methyl state, and found that arginine methylation predominantly exists in the Rme1 form (~84%), and the rest (~16%) are dimethylarginine (including Rme2a and Rme2s) [9]. Guo et al. performed immunoprecipitation and proteomic analysis and found that Rme1 is 3–4 fold more abundant than Rme2a in HCT116 cells [10]. In the *Trypanosoma brucei*, it was reported that methylarginine comprised approximately 10% of the proteome [11], and the proteomic study revealed that the number of peptide fragments containing Rme1 is 8.2-fold of dimethylarginines, and the abundance of Rme2a is 2-fold of Rme2s. A chromatographic analysis of heart/kidney tissue hydrolysates showed that Rme2a level is higher than Rme2s by 5–6 fold [4].

## 2. PRMTs

### 2.1. PRMT Family in Mammalian Cells

**Figure 2:**
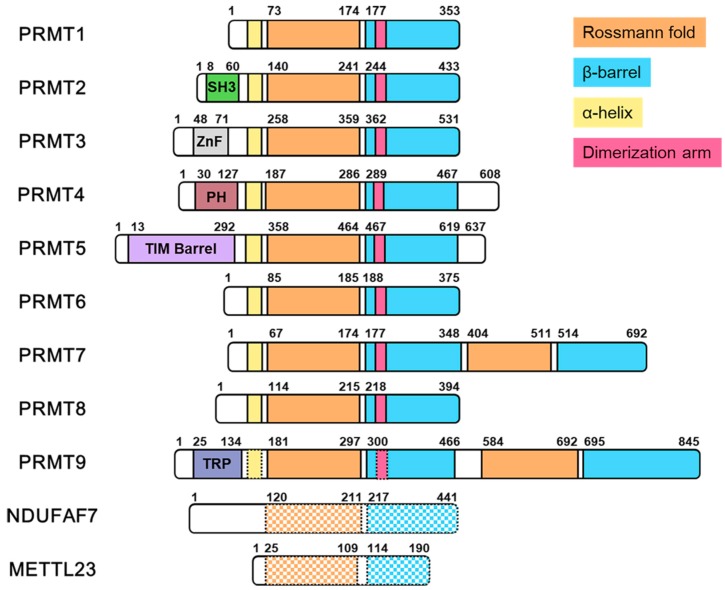
Protein arginine methyltransferases and novel members. For all proteins, the residue numbers are based on human protein sequences. Domains are assigned based on protein alignments within PRMT family members and the examination of the crystal structure data when available. The second Rossmann fold and β-barrel domain in the C-terminal region of PRMT7 is catalytically inactive. The domains of PRMT9 are assigned based on protein alignment alone since crystal structural data is unavailable. The dashed lined α-helix and dimerization arm region in PRMT9 indicates that this is expected, but there is a lack of crystal structure data to confirm this. The dashed, checkerboard pattern in the domains of NDUFAF7 and METTL23 indicates that there is limited protein sequence similarity when compared with the Rossmann fold and β-barrel domains of the PRMTs by sequence alignment. The protein sequences can be found in UniProt: PRMT1, Q99873-3; PRMT2, P55345-1; PRMT3, O60678-1; PRMT4/CARM1, Q86X55-3; PRMT5, O14744-1; PRMT6, Q96LA8-1; PRMT7, Q9NVM4-1; PRMT8, Q9NR22-1; PRMT9, Q6P2P2-1; NDUFAF7, Q7L592-1; METTL23, Q86XA0-1.

Arginine methylation in mammalian cells is catalyzed by the class I, *S*-adenosyl-l-methionine (SAM or AdoMet)-dependent methyltransferases named protein N-arginine methyltransferases (PRMTs) [12,13,14,15,16]. Mammalian cells have nine PRMTs [17], and all have a highly conserved catalytic core composed of a Rossmann-like fold domain that binds SAM and a β-barrel that supports substrate binding (Figure 2) [18]. Upon the initial binding of SAM and substrate, PRMTs transfer the electrophilic methyl group from SAM to a terminal nitrogen on the guanidinium moiety of arginine residues to produce the monomethylarginine residue, Rme1 [3,6,7,8]. All PRMTs will catalyze this initial methyl transfer. Depending on whether or not the PRMT catalyzes a second methyl transfer reaction defines the type of PRMT. PRMT1, -2, -3, -4 (CARM1), -6, and -8 are identified as type I PRMTs, which produce Rme1 and catalyze a second reaction to produce asymmetric dimethylarginine residue (Rme2a) [7]. In contrast, PRMT5 and PRMT9 produce Rme1 and symmetric dimethylarginine residue (Rme2s), which is characteristic of type II PRMTs [15,19,20,21]. While previously misidentified as a type II PRMT, PRMT7 is the only known type III PRMT in mammalian cells that is limited to producing Rme1 [22,23]. Within the PRMT family, PRMT1 and PRMT5 are the major players involved in catalyzing the bulk of arginine methylation in mammalian cells. Chemically induced knockdown of PRMT1 and PRMT5 in mouse embryonic fibroblast cells significantly reduces Rme2a levels by ~58% and Rme2s levels by ~95%, respectively [13,14,15].

### 2.2. Novel Arginine Methyltransferases

In addition to the nine classic PRMT family members, there have been discoveries of other class I mammalian methyltransferases that catalyze arginine methylation. For example, NADH: ubiquinone oxidoreductase complex assembly factor 7 (NDUFAF7) was identified as class I methyltransferase that is responsible for symmetrically dimethylating a highly conserved Arg-85 in NADH: ubiquinone oxidoreductase core subunit S2 (NDUFS2), which is a subunit of the human mitochondrial complex I [24,25]. Symmetric dimethylation of Arg-85 in NDUFS2 was previously discovered in the 49 kDa subunit of bovine complex I [26]. Methylation of Arg-85 in NDUFS2 appears to result in the release of NDUFAF7 and promotes the protein–protein interaction with NDUFS7 for the pre-assembly of active complex I [25]. Recently, the crystal structure of mitochondrial dysfunction protein A (MidA), an ortholog of NDUFAF7 found in *Dictyostelium discoideum*, was solved [27]. Superimposed crystal structures of the MidA and PRMT5 catalytic cores revealed some structural similarities, and a protein sequence alignment with the PRMT family identified conserved residues for SAH binding (Figure 2) [27]. Mettl23 is another class I methyltransferase that was recently discovered in mouse oocytes to catalyze asymmetric dimethylation of histone H3 Arg-17 (H3R17me2a). Mettl23 catalyzed H3R17me2a was demonstrated to support histone H3 deposition and DNA demethylation for paternal reprogramming [28]. Hatanaka et al. performed a protein alignment of PRMT1-8 with Mettl23, and the results reveal some sequence similarities in the methyltransferase core [28]. Since this is a very recent discovery, it remains to be known the type of structural features that Mettl23 and PRMTs share.

## 3. Regulatory Effects of Arginine Methylation on Protein Recognition and Function

### 3.1. Protein-Nucleic Interactions

Arginine methylation is a common PTM mark that regulates important cellular processes such as RNA processing, gene transcription, DNA damage repair, protein translocation, and signal transduction. At the molecular level, this is achieved by altering physicochemical properties of the arginine residue and structural conformation of substrate proteins [29,30]. Arginine methylation can affect protein-nucleic acid interactions. This is not surprising given that the majority (>60%) of the methylated arginine residues are found in proteins that associate with RNA [31]. Moreover, arginine methylation was observed on all transfer RNA synthetases [2]. Rajpurohit et al. showed arginine methylation reduced the binding capacity of hnRNP protein A1 for single-stranded DNA [32]. Methylation of the RGG domain of Ewing’s sarcoma protein (EWS) by PRMT3 resulted in significant loss of the binding ability of EWS to G-quadruplex DNA and RNA in electrophoretic mobility shift assay [33]. The HIV-1 Tat protein is an essential regulatory protein for HIV proliferation and is methylated in the basic region (residues 47–57). Cheng and coworkers studied the effect of different methylation states at different Arg residue in Tat peptides [34]. The *trans*-activation response element (TAR) RNA binding of the peptides was regulated by the position and methylation state. Overall, either Rme1, Rme2a, or Rme2s weakened the binding with RNA. In particular, asymmetric dimethylation at Arg-53 decreased TAR RNA binding affinity by 3-fold. Some methylation marks slightly increased the affinity or had an intangible effect. Thus far, the effects of arginine methylation on RNA or DNA binding can vary, likely depending on macromolecular contexts [29].

### 3.2. Protein-Protein Interactions

Arginine methylation saliently affects protein–protein interaction. Methylarginine can serve as a docking site to support protein–protein interactions. A number of domains have been reported to recognize methylarginine residues in proteins, especially the Tudor domain. Initial reports of proteins recognizing methylated arginine residues focused on survival of motor neurons protein (SMN), which contains a Tudor domain and is a gene product of neurodegenerative disease spinal muscular atrophy gene [35]. Friesen et al. demonstrated that peptide sequences of the carboxy-terminal of small nuclear ribonucleoprotein–associated protein D1 (SmD1) and SmD3 proteins with Rme2s marks could pull-down SMN, and this protein–protein interaction supported the assembly of the small nuclear ribonucleoprotein (snRNP) core particle [35]. Moreover, inhibition of PRMTs in cells by periodate-oxidized adenosine results in the loss of protein–protein interactions with SMN [35]. Similarly, Brahms et al. also observed that symmetric dimethylation of Sm proteins D1, D3, B/B’, and the Sm-like protein 4 (LSm4) is critical for the protein–protein interaction with SMN [36]. Mutation of E134K in the Tudor domain of SMN abolished co-precipitation of SmD1, D3, B, and Lsm4 proteins with SMN [36]. Hence, symmetric dimethylation was important for mediating the protein–protein interaction between SMN and Sm as well as Lsm proteins.

The first report of an effector that recognized the asymmetric dimethylated arginine residues involved CARM1 and SMN. CARM1 methylates the N-terminal region of transcription factor CA150 (CA150), which contained a proline-glycine-methionine-arginine sequence (PGM motif) that was quite different to the common glycine-arginine rich motifs found in PRMT substrates [37]. Moreover, SMN formed protein–protein interactions with CA150 that was dependent on CARM1 methyltransferase activity [37]. TDRD3, γSpf30, and SPF30 are other Tudor-containing proteins that were discovered to recognize asymmetric dimethylarginine residues. TDRD3 preferentially recognizes H3R17me2a and H4R3me2a over H3R2me2a and H3R17me2s, while γSpf30 and SPF30 exhibit weak recognition of H3R17me2a [38,39]. Also, when compared against other Tudor-containing proteins, TDRD3 was demonstrated to coactivate transcription, while SMN and SPF30 could not in an estrogen response element (ERE) luciferase reporter assay [38]. Moreover, knockdown of TDRD3 followed by 30 min treatment with estradiol results in significantly reduced transcription at the pS2 promoter in MCF7 cells [38]. While TDRD3 is detectable in the cytosol and nucleus, TDRD3 is predominantly (63%) in the cytosol [40]. In contrast to the Tudor-containing proteins SMN and TDRD3, the BRCA1 C-terminal (BRCT) domain in TP53-binding protein 1 (53BP1) and the tumor suppressor BRCA1 were discovered to recognize asymmetrically dimethylated Arg-754 in p300 [41]. Upon DNA damage, CARM1 catalyzed asymmetric dimethylation of Arg-754 in p300 is important for recruiting BRCA1 to the p21 promoter to activate gene transcription and regulate the cell cycle [41]. CARM1 also catalyzes the methylation of multiple arginine residues in paired box protein 7 (Pax7) [42]. Substitution of four Arg residues, targeted by CARM1, for Lys residues in the N-terminal region of Pax7 significantly reduces the interaction of Pax7 for mixed lineage leukemia protein 2 (MLL2), and thereby reduces recruitment of the MLL complex to the myogenic determination factor (Myf5) promoter for transcriptional activation [42]. In comparison to the other effectors that recognize methylated arginines, the C-terminal region of MLL2 does not have a Tudor domain and does not bind with CA150 in a CARM1-dependent fashion like SMN [42].

Based on these examples, some of the effectors that recognize methylated arginine residues seem to be promiscuous while others are specific. For instance, TDRD3 prefers ligands with asymmetrically dimethylated arginine residues [38,39], and SMN demonstrates the ability to recognize ligands with symmetrically and asymmetrically dimethylated arginine residues [35,36,39]. When the structures of both effectors are compared, there appears to be structural features that can explain ligand-binding specificity differences between TDRD3 and SMN. TDRD3 appears to recognize methylated arginine residues with a narrow cage of aromatic residues [39]. These structural features appear to support the planar guanidinium group of Arg and distinguishes TDRD3 from other related Tudor domain containing proteins (e.g., 53BP1) that bind methylated lysine residues [39]. In contrast, the crystal structure of SMN revealed a more spacious opening to the cage of aromatic residues, which may explain the ligand binding promiscuity of SMN that others have observed [39]. Hence, there does appear to be some overlap in specificity among the effectors that recognize methylated arginine residues. We have highlighted just a sample of proteins that recognize symmetric and asymmetric dimethylated arginine residues, and others have thoroughly reviewed this subject area elsewhere [30,43].

### 3.3. Liquid-Liquid Phase Separation

Recently, arginine methylation was found to impact the formation of membrane-less organelles inside cells. The phenomenon of liquid–liquid phase separation (LLPS) is the process through which macromolecules separate into two distinct chemical environments through the formation of membrane-less organelles such as the nucleolus, stress granules, and processing bodies (P-bodies) [44]. LLPS serves to concentrate specific macromolecules while excluding others to provide an environment that modulates biochemical reactions [45]. This process is achieved by electrostatic and/or hydrophobic protein-protein or protein-RNA interactions through low-complexity sequences and intrinsically disordered protein regions [44]. The generation of these membrane-less organelles is a reversible process that is driven by the critical concentration of its components or a temperature threshold that, when exceeded, causes the rapid aggregation of components to form the organelle. The best example of this is seen in stress granules, which assemble and disassemble in a rapid response to stressors. In general, stress conditions block translation initiation, and this leads to a sudden increase of mRNA levels. RNA-binding proteins (RBPs) (e.g., TIA-1, TIA-R, and G3BP1) respond by binding to the accumulating levels of non-translated mRNA [46,47]. This, in addition to non-RNA protein-protein interactions, collectively promote the formation of stress granules [44,48].

Recent advances in the development of *in vitro* studies have demonstrated that these liquid-like granules are able to harden into more viscous liquid and gel-like states [45]. These aggregates have been implicated in certain neurodegenerative disorders such as amyotrophic lateral sclerosis, frontotemporal dementia, and Alzheimer’s disease [45]. Seeing as the interactions that promote LLPS are located in intrinsically disordered regions, and these regions are generally the sites of PTMs, it is reasonable to believe that PTMs may play a role in the modulation of the assembly of granules. These regions have been observed to contain high amounts of RG-FG or RGG/RG repeats in proteins such as Ddx4 [49] and FUS [50], respectively. Methylation of arginine does not affect the overall pKa of the residue; however, it does change the shape and charge distribution while increasing hydrophobicity and decreasing its H-bond donor capability [30]. These changes can have dramatic effects on the intermolecular interactions of arginine residues, particularly π-cation interactions. It has been shown that methylation of Ddx^N1^ in *E. coli* by co-expressed PRMT1 reduced the formation of nuage bodies [49]. Also, Qamar et al. showed that hypomethylated FUS showed an increase in droplet formation and had reduced FUS protein dynamics compared to normal FUS [50]. These droplets were shown to be closer to a hydrogel due to their lack of arginine methylation and subsequently stronger π-cation interactions. Currently, there are no examples where arginine methylation promotes LLPS, and it could indicate that Rme2a in general reduces π-cation interactions and reduces phase separation; however, further experimental evidence is needed to demonstrate this [30].

## 4. ADMA—the Small Molecule Metabolite Product of Arginine Methylation

### 4.1. ADMA Production

Once cellular proteins are hydrolyzed, free methylated arginine amino acids are released in the cytosol, and after extracellular transport, these amino acid molecules circulate in biological fluids and the bloodstream. Following proteolysis, Rme1 residue is released as the small molecule *N^G^*-monomethyl-l-arginine (l-NMMA, MMA), Rme2a residue as the small molecule *N^G^*, *N^G^*-dimethylarginine (asymmetric dimethylarginine, ADMA), and Rme2s residue as the small molecule *N^G^*, *N^G’^*-dimethylarginine (symmetric dimethylarginine, SDMA) (Figure 1). In particular, ADMA has gained extensive attention owing to its relationship with endothelial dysfunction and cardiovascular diseases (see Section 4.4.). To date, a *de novo* synthetic pathway of l-NMMA, ADMA, or SDMA from free l-arginine has not been documented. Therefore, free methylarginines found in the plasma and in cells are thought to be derived solely from protein turnover and degradation of proteins containing methylarginines [4]. Along this biological axis, the amount of methylarginine small molecules inside and outside a cell would be critically dependent on the level of arginine methylation in proteins and the rates of protein turnover. As described above, expression and activity of PRMTs play essential roles in determining the abundance of methylation on arginine residues in proteins. In particular, type I PRMTs dictate the level of Rme2a residues and type II PRMTs dictate the level of Rme2s residues. Importantly, the levels of small molecule methylarginines is negatively regulated by dimethylarginine dimethylaminohydrolase (DDAH) enzymes that hydrolyzes ADMA amino acid into citrulline and dimethylamine (see Section 4.2.). Thus, enzymatic activities of PRMTs and DDAHs counteractively regulate the level of ADMA [51,52,53,54]. Wu et al. showed that a PRMT1 inhibitor increased ADMA concentration in a dose-dependent manner both *in vitro* and in unilateral ureter obstructed mouse kidneys [55]. Elevated levels of ADMA are found to associate with lower DDAH2 and higher PRMT1 in cell and animal models [56,57]. Vice versa, reduced ADMA levels in endothelial cells, plasma, and kidney are accompanied with increasing DDAH2 expression and decreasing PRMT1 expression [58,59]. In addition to endogenous generation, humans obtain the methylarginine molecules l-NMMA, ADMA, and SDMA from a variety of vegetables [60].

### 4.2. ADMA Metabolism and Excretion

**Figure 3:**
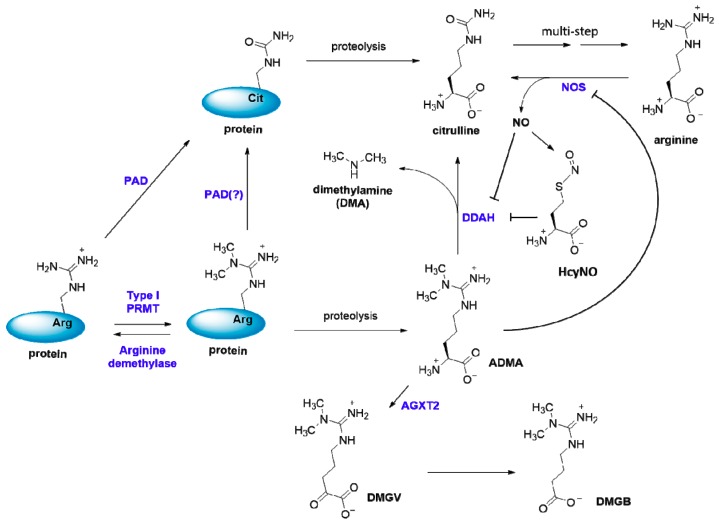
Asymmetric dimethylarginine (ADMA) function and regulation. The free ADMA molecule is produced from proteolysis of proteins containing Rme2a residues. ADMA level is regulated at multiple pathways. At the protein level, type-I PRMTs and potentially PAD and arginine demethylase enzymes regulate the abundance of Rme2a in proteins. Free ADMA is metabolized by DDAH and AGXT2 enzymes. DDAH activity generates citrulline and dimethylamine. AGXT2 activity generates DMGV that is further converted to DMGB. ADMA inhibits nitric oxide (NO) synthesis by competitive binding to nitric oxide synthase (NOS). Homocysteine inhibits DDAH activity via a reactive *S*-nitroso-l-homocysteine adduct.

The primary site of clearance of ADMA from the circulation is the kidney [54,61]; the liver also plays a role [62,63]. Free ADMA was significantly higher in crude kidney extracts than heart extracts [4]. Although methylarginine molecules can be eliminated via renal excretion, ADMA and l-NMMA are also metabolized by dimethylarginine dimethylaminohydrolase (DDAH) into citrulline and dimethylamine/monomethylamine [64]. SDMA cannot be processed by DDAH. A Cys-His-Glu residue triad is involved in DDAH catalysis. The critical cysteine residue (Cys-249) in the active site of DDAH undergoes a nucleophilic attack on the guanidino portion of the ADMA molecule to initiate the breakdown of the scissile C–N bond. Mutation of this cysteine residue to serine renders the enzyme inactive [65]. Importantly, this active-site cysteine is directly regulated by *S*-nitrosylation by nitric oxide (NO) [66]. Cytokine induced expression of nitric oxide synthase (NOS) isoforms in endothelial cells results in *S*-nitrosylation of DDAH, and the *S*-nitrosylation inhibits DDAH activity [66]. DDAH regulation by NO constitutes an interesting feedback mechanism as increased NO generation leads to *S*-nitrosylation DDAH, and its diminished activity, which results in the accumulation of asymmetric dimethylarginine and inhibition of NOS. Of additional interest is that NO is conjugated with homocysteine, another downstream metabolite molecule of biological methylation including arginine methylation, generating a reactive *S*-nitroso-l-homocysteine (HcyNO) to inhibit DDAH activity (Figure 3) [67,68]. This provides another feedback mechanism for the regulation of ADMA and NO levels.

In addition to the primary degradation by DDAH, ADMA can be metabolized with a deamination reaction by alanine-glyoxylate aminotransferase 2 (AGXT2), generating α-keto-δ-(*N^G^,N^G^*-dimethylguanidino)valeric acid (DMGV), which can be further metabolized into γ-(*N^G^,N^G^*-dimethylguanidino)butyric acid (DMGB) [69]. Different from DDAH, AGXT2 has a broader substrate specificity for methylarginines; it can utilize not only ADMA or l-NMMA, but also SDMA, as a substrate. Lentz and colleagues were the first to clone the human AGXT2 gene, and they found that an overexpression of human AGXT2 in mice led to decreases in plasma and tissue levels of ADMA [70]. On the other hand, AGXT2-deficient mice have increased plasma concentrations of both ADMA and SDMA [71]. In accordance, the kidney is the major site of metabolism of ADMA by AGXT2 [69]. There are two mechanisms of renal clearance of ADMA: (1) a direct mechanism wherein ADMA is excreted into the urine unchanged, and (2) an indirect mechanism in which ADMA is first metabolized by AGXT2 and then excreted into the urine as DMGV or DMGB. Moreover, it appears that the majority of ADMA is metabolized. In a metabolism study with rats that were administered radiolabelled ADMA, the fraction of unchanged ADMA represented approximately 35% of the recovered metabolites in the urine, and this represented 4.5% of the total radiolablled ADMA that was administered [69]. The clearance of ADMA through the AGXT2-mediated mechanism appears to be more efficient than the direct excretion of ADMA [72]. Physiologically, AGXT2 could be essential to protect animals from ADMA-mediated impairment of NO production [71,72].

Another possible mechanism of regulation of ADMA could be through PTMs in protein substrates. It is well known that arginine residues can be deiminated by the enzyme family of protein (or peptidyl) arginine deiminases (PADs) which convert arginine residue to citrulline residue [3]. One could imagine that the level of ADMA may decrease if PADs deiminate methylarginine residue at the protein level. However, reported data suggest that Rme2a residue cannot be deiminated by PAD4 and Rme1 residue can only be weakly deiminated by PAD4 [73,74,75]. Since most of these measurements were done on purified enzymes and substrates, a more systematic investigation of all the PAD members under *in vivo* scenarios will be of great value to confirm these data. It would be interesting to test if any PAD overexpression or knockdown can affect l-NMMA, ADMA, and SDMA levels in cells and in plasma.

Last but not least, ADMA may be regulated by the demethylation of methylated arginine residues in proteins. 2-Oxoglutarate-dependent JmjC-domain-containing protein 6 (JMJD6) was the first reported arginine demethylase, and it targets histone marks H4R3me2s, H4R3me2a, and H3R2me2a [76]. Since then, others have reported additional JMJD6 demethylation targets such as ERα, TRAF6, G3BP1, HSP70, and RHA [77,78,79,80,81]. However, different research groups report that JMJD6 mainly catalyzes lysine hydroxylation, and there was difficulty in reproducing the dimethyl-arginine demethylation reaction with the H4 and H3 peptides [82,83,84,85,86]. Recently, the lysine demethylase JMJD1B was discovered as an arginine demethylase of H4R3me1 and H4R3me2s [87]. Other JmjC-containing members of the lysine demethylases (KDM) have been discovered (KDM4A/4E, KDM3A, KDM5C, KDM6B) to possess arginine demethylase activity based on NMR and mass spectrometry analysis [88]. For more information, detailed reviews of the arginine demethylase enzymes were recently published [89,90]. If approved as *bona fide* arginine demethylases, these KDM enzymes are capable of decreasing methylarginine levels in proteins, thereby decreasing free methylarginine amino acids.

### 4.3. Inhibition of NOS by ADMA

Endothelial cells of the inner vessels use the enzymatic activity of nitric oxide synthase (NOS) to convert a molecule of l-arginine into NO, which diffuses from endothelial cells to the smooth muscle cells, leading to blood vessel vasodilation and decreased blood pressure [30]. Thus, NO acts as an important endogenous vasodilator. At the molecular level, ADMA is a structural analog of l-arginine and therefore can compete with l-arginine for binding to the NOS catalytic site to inhibit NO synthesis. Interestingly, l-NMMA and ADMA inhibit all 3 isoforms of NOS with approximately equivalent potency, while SDMA does not inhibit NOS [54,91,92]. Although both l-NMMA and ADMA inhibit NOS activity, ADMA is considered to be a more physiologically relevant NOS inhibitor than l-NMMA owing to its higher concentration in plasma. Consistent with the ADMA hydrolysis activity of DDAH, alterations in DDAH expression appreciably changes ADMA and NO concentrations. DDAH-1 activity leads to accumulation of ADMA and reduction in NO signaling [93]. NOS activity and NO production are increased in response to overexpression of human DDAH-1 in cultured murine endothelial cells [94]. Moreover, NOS activity and urinary nitrogen oxides increase by at least 2-fold while plasma ADMA is reduced by just over 2-fold in transgenic mice models expressing human DDAH-1 [94].

### 4.4. Physiological Impact of ADMA

Endogenous ADMA is usually excreted in the urine. However, in patients with acute or chronic renal failure, who have limited or no urine output, elimination of ADMA is blocked and circulating concentrations of ADMA rise, which may interfere with NO synthesis [95,96]. The scenario is further acerbated by the significantly reduced plasma arginine concentrations in patients with renal failure in comparison to healthy patients [95]. Of pathophysiological significance, elevated circulating plasma concentrations of ADMA provide a marker of risk for a wide range of endothelial dysfunction and renal and cardiovascular disorders, such as renal failure, chronic obstructive pulmonary disease (COPD) such as pulmonary arterial hypertension [97], pulmonary fibrosis, rheumatoid arthritis (RA) [98], increased cardiovascular morbidity and mortality, diabetes, heart failure, and elevated systemic blood pressure [54,99,100]. High plasma ADMA was correlated with high all-cause mortality and late mortality in renal transplant recipients. In contrary, urinary excretion of ADMA and SDMA is inversely associated with cardiovascular disease (CVD) and all-cause mortality [101]. Exogenous treatment of human monocytoid cells THP-1 with micromolar concentrations of ADMA significantly upregulates the levels of IL-8, TNF-α, MCP-1, chemokine receptors CCR_2_ and CXCR_2_, activation of NF-κB, and promotes monocytic binding to human umbilical vein endothelial cells [57]. ADMA could also be a biomarker for insulin resistance [102] and a sleep disorder caused by misregulation of DDAH expression [103].

Although accumulation of plasma ADMA correlates with impaired NO synthesis and contributes to the hypertension and immune dysfunction associated with chronic renal failure, it appears that the (patho)physiological impact of ADMA does not take place in plasma [91]. Plasma arginine concentrations are in the range of ~100 to 200 µM, but the plasma ADMA concentrations are much less, 0.3–1.0 µM at a typical level [54,61,101,104,105,106,107]. Thus, in the circulating bloodstream, ADMA is not competent enough against arginine for receptor binding. On the other hand, the intracellular concentrations of ADMA appear to be much higher than extracellular environment [91,92,106]. In one case, concentrations of l-NMMA and ADMA were 5.2 and 5.0 µM in the endothelial cells [108]. Indeed, a recent study showed that effect of decreased ADMA correlates with enhanced NO concentration locally in renal tubules but not in the whole UUO kidney [55].

### 4.5. Medicinal Significance of PRMT Inhibitors in ADMA-caused Cardiovascular Disorders

Given that ADMA is derived as the end product of type I PRMT activity, especially the major type I enzyme PRMT1, inhibition of type I PRMT enzymes might be of therapeutic value in counteracting the problem of ADMA. PRMT inhibitors (PRMTi) are currently extensively applied in cancer treatment. The therapeutic significance of PRMTi in cardiovascular pathology will need to be explored. The anti-hypertension drug metformin appears to decrease ADMA levels. However, in our hands, we did not observe any inhibitory effect of metformin on the activity of PRMT1 (unpublished data). Thus, metformin does not seem to act through PRMT inhibition. So far, a plethora of PRMT inhibitors have been reported [8,109,110,111]. We anticipate that potent PRMT1 inhibitors will be studied for their potential therapeutic value in counteracting hypertension and endothelial dysfunction. A recent study hints at this therapeutic potential [55]. Since PRMT5 (the major type II enzyme) competes with PRMT1 for the pool of Rme1 residues in proteins, knockdown of PRMT5 may increase the abundance of Rme1 sites in proteins that are substrates of PRMT1 for Rme2a formation [14]. In this perspective, we predict that PRMT5 inhibitors might increase the level of ADMA and lead to cardiovascular side effects.

## 5. Perspective

The biological axis of arginine methylation-ADMA-NO pathway showcases a paradigm of how protein PTMs are intricately coupled with metabolic regulation. On one hand, PRMTs can directly affect cell biology by modulating their protein substrates. For instance, PRMT1 promotes hyperglycemia by methylating the transcriptional factor forkhead box O1 (FOXO1) and thus increasing its nuclear retention and the transcriptional activity [112]. On the other hand, PRMTs impact on (patho)physiology via its far end metabolite product, ADMA. So far arginine methylation appears to be the most renowned PTM that prompts a dramatic physiological and pathophysiological impact through metabolic end products. There are many intriguing questions that are not fully addressed in the ADMA biology, which could be worthy areas of investigation in the field. We herein list just a few of them based on our opinions.

Protein degradation occurs through either the proteasome or the lysosome. Yet, it remains to be determined which degradation pathway predominately contributes to the small molecule ADMA formation. Also, it is not clear what proteins contribute to the accumulation of ADMA. Related, which tissues are the major source of plasma ADMA levels? The known abundant proteins in cells are histones, actin, and tubulin. However, the richness of arginine methylation in these proteins seems to be very limited. In plasma, albumin is an abundant protein. Does albumin contain a lot of Rme2a residues that would serve as precursors to the ADMA metabolite through albumin degradation? Except through PRMT-mediated methylation, dynamic regulation of ADMA via protein arginine citrullination, demethylation, and other types of protein arginine modifications is not clear.

Although NOS is the most studied target for ADMA effect, it is not clear if ADMA has additional significant biological targets. From a pharmacological point of view, a drug’s action of mechanism is often dependent on its concentrations. At increased concentrations of ADMA, it could bind to targets additional to NOS. Moreover, does ADMA have any beneficial effect, or is it just an unfortunate side-product resulted from protein arginine methylation?

Given that the abundance of methylarginine residues in proteins is in the order of l-NMMA>ADMA>SDMA, in principle the level of free methyl-arginine molecules in the plasma should follow the same order, but studies have shown that this is not the case. Instead, concentrations of free ADMA are greater than that of l-NMMA both in the plasma and in the cells [95,113,114]. The disproportionate correlation of the abundance of Rme1 and Rme2a in proteins versus the l-NMMA/ADMA ratios in cytosol and in plasma is poorly unexplained. Possibly, l-NMMA is more actively processed than ADMA by DDAH in cells. Also, the cationic amino acid transporter (CAT) may transport l-NMMA, ADMA, and SDMA across the plasma membranes at differential efficiencies [114]. Except for ADMA, pathophysiological significance of l-NMMA and SDMA were less appreciated. Plasma ADMA and SDMA concentrations appear to be approximately equivalent [114]. Also, l-NMMA and ADMA inhibit all 3 isoforms of NOS with approximately equipotency [54]. It remains to be determined if there is any significant biological activities of cellular or plasma l-NMMA and SDMA molecules. In this regard, studies suggest that SDMA interferes with CAT-mediated l-arginine transportation [96]. Furthermore, citrulline is generated by DDAH activity of l-NMMA and ADMA. Does citrulline have any significant activity in biological regulation?

From a broader perspective of protein methylation and the production of downstream methylated metabolites, the field of protein arginine methylation on metabolic biology is shared with protein lysine methylation. Proteins with trimethylated lysine residues can undergo protein degradation to generate the small molecule, 6-N-trimethyllysine (TML). TML is the precursor for the multi-step synthesis of carnitine, which acts as an acyl group carrier in fatty acid oxidation [115]. Besides the canonical function of PTMs regulating protein properties, the regulation of cell biology by protein PTMs via end metabolite molecules is a still largely an untapped area of research.

## Abbreviations

53BP1	TP53-binding protein 1
ADMA	The small molecule metabolite *N^G^*, *N^G^*-dimethylarginine (asymmetric dimethylarginine)
AGXT2	Alanine-glyoxylate aminotransferase 2
BRCA1	Breast cancer type 1 susceptibility protein
BRCT	BRCA1 C-terminal domain
CA150	Transcription factor CA150
CARM1	Coactivator associated arginine methyltransferase 1
CCR_2_	C-C chemokine receptor type 2
CVD	Cardiovascular disease
CXCR_2_	C-X-C chemokine receptor type 2
DDAH	Dimethylarginine dimethylaminohydrolase
Ddx4	DEAD box protein 4
DMGB	γ-(*N^G^,N^G^*-dimethylguanidino)butyric acid
DMGV	α-keto-δ-(*N^G^*,*N^G^*-dimethylguanidino)valeric acid
ERα	Estrogen receptor alpha
ERE	Estrogen response element
EWS	Ewing’s sarcoma protein
FOXO1	Forkhead box protein O1
FUS	Fused in sarcoma
G3BP1	Ras-GTPase activating SH3 domain-binding protein 1
HcyNO	*S*-nitroso-l-homocysteine
HSP70	Heat-shock protein of 70 kDa
IL-8	Interleukin-8
JMJD	JmjC-domain-containing protein
LLPS	Liquid-liquid phase separation
l-NMMA	The small molecule *N^G^*-monomethyl-l-arginine (MMA)
Lsm	Sm-like protein
MCP-1	Monocyte chemoattractant protein-1
Mettl23	Methyltransferase-like protein 23
MidA	Mitochondrial dysfunction protein A
MLL	Mixed lineage leukemia protein
NDUFAF7	NADH: ubiquinone oxidoreductase complex assembly factor 7
NDUFS2	NADH: ubiquinone oxidoreductase core subunit S2
NF-κB	Nuclear factor of kappa light polypeptide gene enhancer in B-cells 1
NO	Nitric oxide
NOS	Nitric oxide synthase
Pax7	Paired box protein 7
P-bodies	Processing bodies
p300	Histone acetyltransferase p300
PRMT	Protein N-arginine methyltransferase
PRMTi	PRMT inhibitor
PTM	Post-translational modification
RBP	RNA-binding protein
RHA	RNA helicase A
Rme1	Monomethylarginine residue in proteins
Rme2a	Asymmetric dimethylarginine residue in proteins
Rme2s	Symmetric dimethylarginine residue in proteins
SAH	*S*-adenosyl-l-homocysteine
SAM	*S*-adenosyl-l-methionine (AdoMet)
SDMA	The small molecule *N^G^*, *N^’G^*-dimethylarginine (symmetric dimethylarginine)
Sm	“Smith,” small nuclear ribonucleoprotein-associated protein
SMN	Survival of motor neurons protein
snRNP	Small nuclear ribonucleoprotein
SPF30	Survival of motor neuron-related-splicing factor 30
TAR	*trans*-activation response element
TDRD	Tudor domain-containing protein
TIA-1	T-cell intracellular antigen-1
TIA-R	TIA-1 related protein
TNF-α	Tumor necrosis factor alpha
TML	6-N-trimethyllysine
TRAF 6	TNF receptor-associated factor 6
UUO	Unilateral ureter obstruction
